# Genome-wide association study of BNT162b2 vaccine-related myocarditis identifies potential predisposing functional areas in Hong Kong adolescents

**DOI:** 10.1186/s12863-024-01238-6

**Published:** 2024-06-06

**Authors:** Chun Hing She, Hing Wai Tsang, Xingtian Yang, Sabrina SL Tsao, Clara SM Tang, Sophelia HS Chan, Mike YW Kwan, Gilbert T Chua, Wanling Yang, Patrick Ip

**Affiliations:** 1 Department of Paediatrics and Adolescent Medicine, Li Ka Shing Faculty of Medicine, The University of Hong Kong, Pokfulam, Hong Kong SAR China; 2 Department of Surgery, Li Ka Shing Faculty of Medicine, The University of Hong Kong, Pokfulam, Hong Kong SAR China

**Keywords:** Vaccine-induced myocarditis, BNT162b2 vaccine, Genetic risk predisposition, Clustering, Vaccine side effect

## Abstract

Vaccine-related myocarditis associated with the BNT162b2 vaccine is a rare complication, with a higher risk observed in male adolescents. However, the contribution of genetic factors to this condition remains uncertain. In this study, we conducted a comprehensive genetic association analysis in a cohort of 43 Hong Kong Chinese adolescents who were diagnosed with myocarditis shortly after receiving the BNT162b2 mRNA COVID-19 vaccine. A comparison of whole-genome sequencing data was performed between the confirmed myocarditis cases and a control group of 481 healthy individuals. To narrow down potential genomic regions of interest, we employed a novel clustering approach called ClusterAnalyzer, which prioritised 2,182 genomic regions overlapping with 1,499 genes for further investigation. Our pathway analysis revealed significant enrichment of these genes in functions related to cardiac conduction, ion channel activity, plasma membrane adhesion, and axonogenesis. These findings suggest a potential genetic predisposition in these specific functional areas that may contribute to the observed side effect of the vaccine. Nevertheless, further validation through larger-scale studies is imperative to confirm these findings. Given the increasing prominence of mRNA vaccines as a promising strategy for disease prevention and treatment, understanding the genetic factors associated with vaccine-related myocarditis assumes paramount importance. Our study provides valuable insights that significantly advance our understanding in this regard and serve as a valuable foundation for future research endeavours in this field.

**Supplementary Information:**

The online version contains supplementary material available at 10.1186/s12863-024-01238-6.

## Introduction

Since the COVID-19 outbreak, different types of vaccines have been developed and swiftly approved for emergency use worldwide by the US Food and Drug Administration (FDA) [1–3]. As early as April 2021, various platform-specific adverse effects have been reported following the administration of mRNA vaccines (Pfizer-BioNTech (BNT162b2) and Moderna (mRNA-1273) vaccines) through surveillance programs globally. These adverse effects include clotting disorders, Guillian-Barre syndrome, leukocytoclastic vasculitis [4–6], as well as acute myocarditis and pericarditis [7, 8]. Comparing the incidence rates of vaccine-induced myocarditis in Hong Kong and the US, the incidence rate in Hong Kong was at 18.52 per 100,000 adolescents (age 12–17) [9] which is much higher than that of 6.28 per 100,000 in the US [10]. Furthermore, the incidence appeared to be higher in males than females, in adolescents (aged 12–29) compared to adults, and in individuals receiving the second dose compared to the first dose [9, 11–16]. Most cases of BNT162b2-associated myocarditis presented with mild symptoms such as chest pain, which typically required either no treatment or the use of nonsteroidal anti-inflammatory drugs (NSAIDs) [9].

Messenger RNA (mRNA) vaccines introduce a small piece of genetic material called mRNA into host cells. In the case of BNT162b2 vaccines, the mRNA encoding the spike protein (S protein) of the virus is administered intramuscularly [17]. The mRNA delivered via lipid nanoparticles is taken up by host cells; it then escapes into host cell cytoplasm and instructs the production of the S protein [17]. S proteins produced by the host are expressed on the surface of host cells, and subsequently recognized, processed and presented by antigen-presenting cells to induce adaptive immune response via expansion of S protein-specific T cells (CD4^+^ and CD8^+^) [17]. Consequently, the generation of memory B cells and T cells enables a faster and stronger immune response on future insults.

While the etiology of vaccine-induced myocarditis remains unclear, molecular mimicry against S protein, where antibodies against SARS-CoV-2 cross-react with host tissue proteins, was believed to trigger the autoimmune response of vaccine-induced myocarditis [18]. The fact that subjects are more likely to develop myocarditis after second dose might be due to the fact that the first dose sensitized the immune system, and the second dose activates it [18]. High levels of testosterone were suspected to cause the higher incidence rate in males, possibly by promoting aggressive helper T cell response and downregulating anti-inflammatory immune cells [18]. The involvement of autoimmunity in mediating this complication motivated the hypothesis that genetic variations regulating the activities of autoimmune cells modulates disease susceptibility of vaccine-induced myocarditis. Recent identification of disease susceptibility loci in *HLA* (DRB1*14:01, DRB1*15:03, and motifs in HLA-A (Leu62 and Gln63)) and *KIR* (*KIR2DL5B(−)/KIR2DS3(+)/KIR2DS5(−)/KIR2DS4del(+)*) [19, 20] suggested the involvement of T cells and NK cells in inducing myocarditis post vaccination, demonstrating the role of genetic predisposition in mediating susceptibility of vaccine-induced myocarditis. To our knowledge, most genetic studies in this field have taken a similar candidate gene approach, this however is biased towards immune-related genes. An unbiased genome-wide approach with the potential of uncovering disease susceptibility loci beyond immune-related genes could greatly impact personalized care in vaccine administration. This allows healthcare professionals to develop targeted screening strategies, prioritize monitoring for high-risk individuals, and implement early interventions. This personalized approach would enhance patient safety, improve outcomes, and contribute to the ongoing development of safer vaccines.

The important role of genetics can also be seen from a case of vaccine-induced myocarditis in 13-year-old monozygotic twins, which indicated a high degree of phenotypic similarity [21]. Studying rare variant associations would require a large cohort, necessitating ongoing recruitment and international collaboration. Common variants on the other hand, can be studied as clusters by linkage to enhance power in smaller cohorts. As a local effort to investigate the genetic role in vaccine-induced myocarditis, the current study aims to systematically analyze common variant associations of BNT162b2 vaccine-related myocarditis in a cohort of Hong Kong adolescent patients, leading to a hypothesis that disease susceptibility loci related to vaccine-induced myocarditis can be uncovered by using an unbiased genome-wide approach.

## Methods and materials

### Subject recruitment

This study was conducted on a cohort of confirmed cases reported earlier by our team [9, 22]. Individuals who received the mRNA COVID-19 vaccine consented to link their electronic health records from the Hospital Authority (HA) to their vaccination records through the COVID-19 vaccines Adverse events Response and Evaluation (CARE) programme. Participants aged 12–17 years with suspected post-vaccine acute myocarditis, having received the 1st, 2nd, or 3rd dose of the mRNA COVID-19 vaccine within 14 days before admission to a Hong Kong Hospital Authority (HA) hospital, were included in this study. A series of cardiological investigations were monitored for the admitted patients, including measuring serum cardiac troponin T, electrocardiograms (ECGs) and echocardiograms for the admitted patients. These cases were confirmed according to the criteria suggested by the Brighton Collaboration Case Definition of Myocarditis and Pericarditis and managed by the study team following the Hong Kong Paediatrics Investigation Protocol for Comirnaty-related Myocarditis/Pericarditis [23]. The exclusion criteria followed the epidemiological study published earlier by our group [9].

WGS data of 375 Hong Kong Chinese from a study of Hirschsprung’s disease conducted at The University of Hong Kong [24] and 106 healthy Hong Kong Chinese parents from another study on biliary atresia (unpublished) were used as controls for the association analysis. Allosomal data were not available for the dataset of 375 subjects. Importantly, since all control data were collected before the COVID-19 pandemic, none of the controls had a known diagnosis of vaccine-related myocarditis. To date, there have been no reported mechanistic or pathogenic crosstalk between the phenotypes of the controls (Hirschsprung’s disease and biliary atresia) and vaccine-related myocarditis, thus the sequencing data could be adopted as control dataset in conducting the association analysis in this study.

### Whole genome sequencing, variant calling, and association analysis

Whole genome sequencing was performed on genomic DNA extracted from whole blood samples of 43 patients with vaccine-induced myocarditis. The sequencing was conducted using the DNBseq platform with paired-end reads (2 × 150 bp). However, 8 of the 43 samples had inconsistent FASTQ file formats and were excluded from downstream analysis. The raw reads from the remaining 35 samples were first trimmed using trimgalore v0.6.6 [25] to remove adapter sequences and low-quality bases. The trimmed reads were then aligned to the UCSC human reference genome build hg19 using bwa v0.7.17 [26]. The average depth of coverage was estimated to be 30X. Variant calling was performed using DeepVariant v1.4.0, and joint genotyping was conducted using GLnexus v1.4.1 [27] to generate the Variant Call Format (VCF) file for the cases. Variants from low coverage regions (DP < 10) were excluded to minimize variant calling errors.

The control dataset was obtained from other sources as previously described, and only genotype data in VCF format were available. The average depths of coverage were estimated to be approximately 30X for the control subjects from both the biliary atresia study and the Hirschsprung’s disease study. Three control subjects were found being related and therefore excluded from the analysis.

Quality control procedures were implemented using PLINK v1.90b5.3 [28] and an overview of these procedures was illustrated in Figure S1. Non-autosomal SNPs and SNPs with genotype missingness greater than 10% or sample missingness greater than 10% were excluded. Additionally, based on the control VCF, SNPs with a minor allele frequency less than 1% or deviating from Hardy-Weinberg equilibrium (*p* < 0.0001) were excluded. The association analysis was performed on the remaining 6,454,355 SNPs using an additive model to test their association with vaccine-induced myocarditis. Population stratification was adjusted using principal component analysis (PCA), and the first six principal components, along with sex, were included as covariates in the association analysis (Figure S2 and Figure S3).

### ClusterAnalyzer to identify regions with consistent association P values among SNPs in intermediate to high LD

**Fig. 1:**
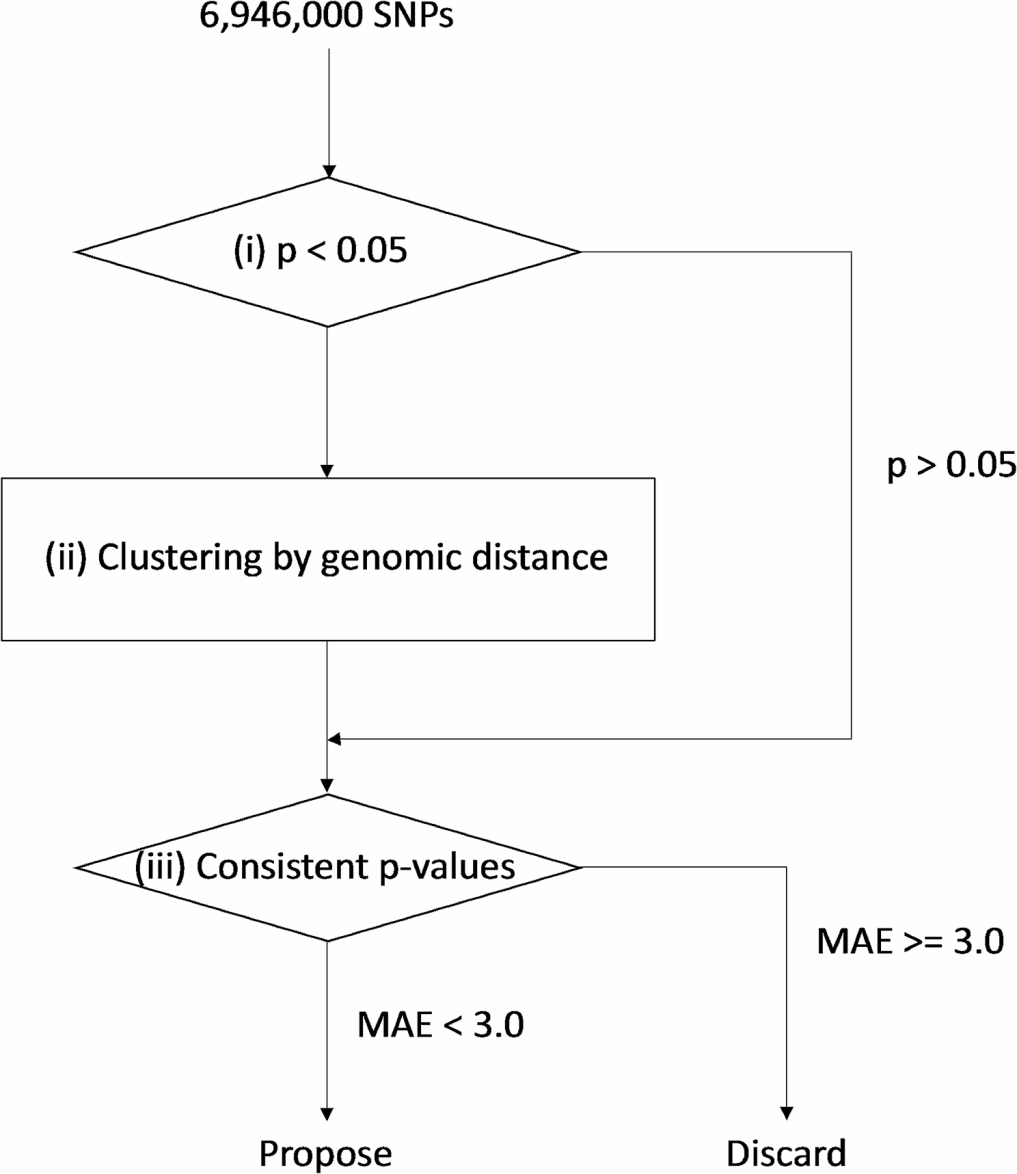
Schematics of ClusterAnalyzer. Based on summary statistics of SNPs tested for BNT162b2 vaccine-related myocarditis association, (i) SNPs with *p* < 0.05 were selected and (ii) clustered by genomic distance. P-value consistencies were evaluated for each signal within each cluster and quantified as Mean Absolute Error (MAEs). Clusters containing at least one signal with an MAE < 3.0 were prioritized

Based on the summary statistics of SNP associations for vaccine-related myocarditis, the ClusterAnalyzer was designed to prioritize genomic regions. (Fig. 1) Genomic regions are defined as non-overlapping sets of SNPs with a *p* < 0.05 (significant SNPs) located within 5 kb of one another. To ensure computational efficiency, only regions with a minimum of 10 significant SNPs were considered.

For each genomic region, SNPs in linkage equilibrium (LD; R^2^ > 0.4) were categorized based on the direction of the effect (OR > 1 and OR < 1), enabling the separate evaluation of p-value consistencies for independent signals. Within each group, p-value consistency was determined by the deviation from the line shown in (A) and quantified as Mean Absolute Errors (MAEs, averaged residuals of *K* SNPs in the group) in (B). Here, *s* represents -log(P) of each SNPs, *s*_max_ represents -log(P) of the top SNP, and *r*^*2*^ represents LD(R^2^) between the top SNP and other SNPs in the group. Larger MAE indicated poorer p-value consistencies and are thus more likely to be false signals.

$$\left(\varvec{A}\right) \widehat{s}={s}_{max}\times {r}^{2}$$

$$\left(\varvec{B}\right) MAE=\frac{\sum _{k}^{K}|{s}_{k}-\widehat{{s}_{k}}|}{K}$$

Utilizing a user-defined threshold for MAE, a summary of prioritized genomic regions is presented in TSV format. Additionally, GWAS summary statistics of variants within the chosen regions are reported in PLINK format.

### Gene set enrichment analysis

The prioritized genomic regions were extended by 5 kb on each end, and the genes within these regions were tabulated using hg19 gene regions obtained from the UCSC database (http://api.genome.ucsc.edu/getData/track? genome=hg19;track=refGene) and processed with pybedtools v0.9.1 [29]. The list of annotated genes in these regions was submitted to the g: Profiler Gene Ontology Statistics (g:GOSt) web server (https://biit.cs.ut.ee/gprofiler/gost) [30] for functional annotation. Gene set enrichment analysis was conducted based on Gene Ontology molecular function (GO:MF) and Gene Ontology biological process (GO:BP) manual annotations. The reference gene sets were derived from “gprofiler_full_hsapiens.name.gmt” and “gprofiler_full_hsapiens.ENSG.gmt.” Default statistical thresholds were used, except for “term size,” which refers to the number of genes enriched in a particular pathway. “Term size” was set within a range of 5-350 to exclude large pathways with limited interpretive value and small pathways with reduced statistical power [31]. Identified biological processes and molecular functions were clustered using the EnrichmentMap and AutoAnnotate applications (MCL Cluster) in Cytoscape v3.10.1 [32].

### Statistical analysis and data visualization

Quality control procedures and LD computation were executed using PLINK v1.90b5.3 [28]. Manhattan plots and QQ-plots were generated with the Python package qmplot v0.3.2 (https://github.com/ShujiaHuang/qmplot), seaborn v0.12.1, and matplotlib v3.6.2. Regional association plots of selected regions were produced using LocusZoom v0.14.0 (http://locuszoom.sph.umich.edu/). ClusterAnalyzer and other statistical analyses were implemented in Python.

## Results

Forty-three Hong Kong Chinese adolescents, aged between 12 and 17 years, with good past health were diagnosed with vaccine-related myocarditis between July 2021 to June 2022 after a median of 3 days after receiving the BNT162b2 vaccine (Comirnaty). The patient cohorts composed of 38 male patients (88.4%) and 5 female patients (12.6%). Majority (81.4%) presented with myocarditis after receiving the 2nd dose of the vaccine. Table 1 provides a summary of the demographic information of the recruited subjects. Detailed clinical background of the confirmed cases was shown in our earlier published studies [9, 22]. The confirmed cases presented common cardiac symptoms including chest pain and palpitations. Abnormal ECGs and echocardiogram were observed with an elevated serum cTnT concentration in patients. Overall, these findings suggested that the confirmed cases exhibited an over-reacted host response to the BNT162b2 vaccine and experiencing the adverse cardiac events.

**Table 1:** Demographics of the post-mRNA COVID-19 vaccinated acute myocarditis and healthy control

		BNT162b2 vaccine-related myocarditis cases (*N* = 43)	Effective BNT162b2 vaccine-related myocarditis cases (*N* = 35)	Healthy Control (*N* = 481)	Effective healthy control (*N* = 478)
Sex (%)/n	Male	88.4 (38)	88.6 (31)	72.6 (349)	72.6 (347)
	Female	12.6 (5)	11.4 (4)	27.4 (132)	27.4 (131)
Onset Doses (%)/n	1st	16.3 (7)	17.1 (6)	--	--
	2nd	81.4 (35)	80.0 (28)	--	--
	3rd	2.3 (1)	2.9 (1)	--	--
Median days of hospital admission from the last dose/ range		3 (1–14)		--	
Sample Collection Period		July 2021 - June 2022		--	

**Fig. 2:**
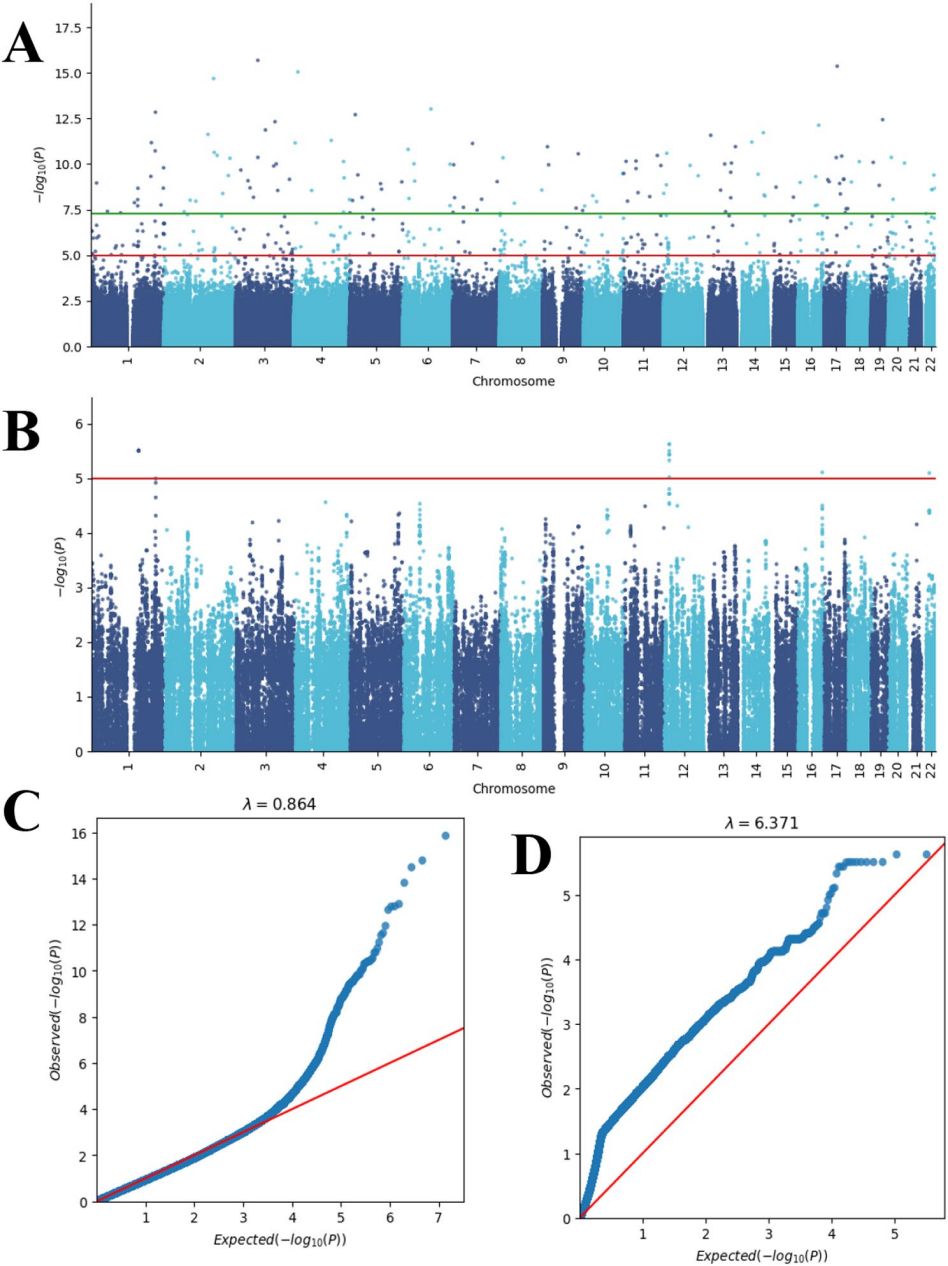
Manhattan and quantile-quantile (QQ) plots. Manhattan plot of **(A)** initial SNP associations and **(B)** prioritized SNP associations using ClusterAnalyzer. The horizontal axis in each plot represents the SNP location from chromosome 1 to chromosome 22, while the vertical axis represents significance level in negative logarithm. The red line corresponds to *p* = 1.0 × 10^− 5^. Green line corresponds to *p* = 5.0 × 10^− 8^. QQ plots are also provided for **(C)** initial SNP associations and **(D)** prioritized SNP associations

The effective 35 BNT162b2 vaccine-related myocarditis patients and 478 Hong Kong Chinese controls sequencing data were analyzed. Using an additive model, we tested the association of 6,454,355 SNPs related to vaccine-related myocarditis. Multiple SNPs reached genome-wide significance (*p* < 5 × 10^− 8^; Fig. 2A), but only a small portion of SNPs formed stacks across the genome. In light of this, we designed a clustering approach to partially remove likely false positive signals due to the genotyping errors.

**Fig. 3:**
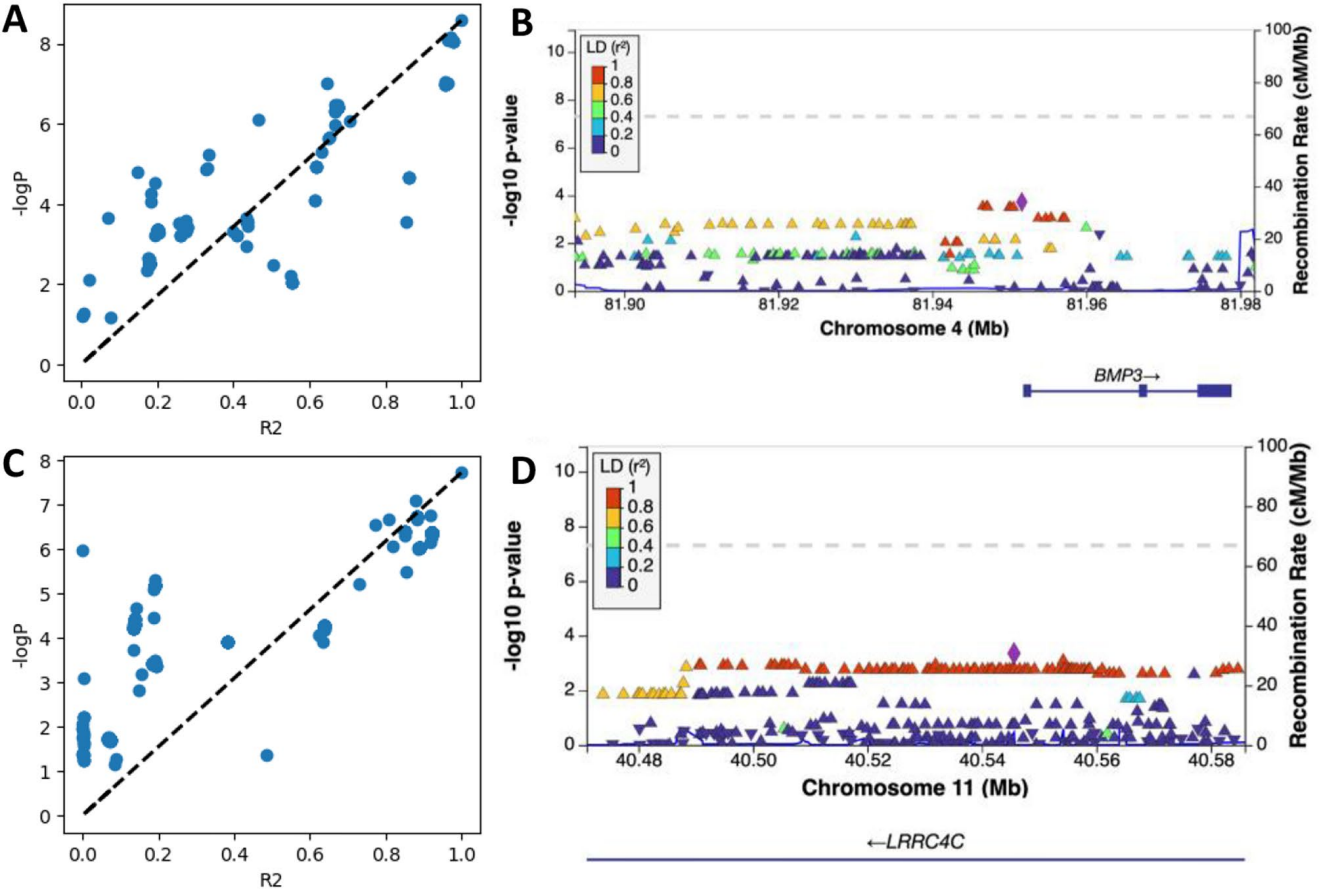
Linkage-association plots and regional association plots generated by LocusZoom for chr4:81,892,037–81,965,272 (**A**, **B**) and chr11:40,473,809 − 40,585,162 (**C**, **D**). SNPs with different direction of Odds Ratio (OR) compared to the top SNP are not shown. Dotted lines represent predicted log association P values based on Linkage Disequilibrium (LD, R^2^)

By setting a mean absolute error (MAE) threshold of 3.0 for the ClusterAnalyzer tool, we narrowed down the list of SNPs to 2,182 genomic regions, which contained 162,578 SNPs (Fig. 1). The average coverage of SNPs in these prioritized genomic regions was estimated at 27X, comparable to the genome average, indicating minimal bias towards sequence coverage. After applying the ClusterAnalyzer, the genomic SNPs appeared more organized as stacks (Fig. 2A and B), and the genome inflation factor has increased significantly from 0.864 to 6.371 (Fig. 2C and D). The tool effectively narrowed down the list of SNPs to a smaller subset, namely 2.5% of all SNPs. Two of the prioritized regions most enriched in significant SNPs were chr4:81,892,037–81,965,272 and chr11:40,473,809 − 49,585,162 (hg19; Fig. 3). Linkage-association plots showed linked SNPs have comparable log association values (within 3 logs units and adhered to the fitted line; Fig. 3), which is evidence of likely true signal. These regions localized to Bone Morphogenetic Protein 3 (*BMP3*; Fig. 3B) and Leucine-Rich Repeat Containing 4 C (*LRRC4C*; Fig. 3D), suggesting a potential connection between these genomic regions and vaccine-related myocarditis.

**Fig. 4:**
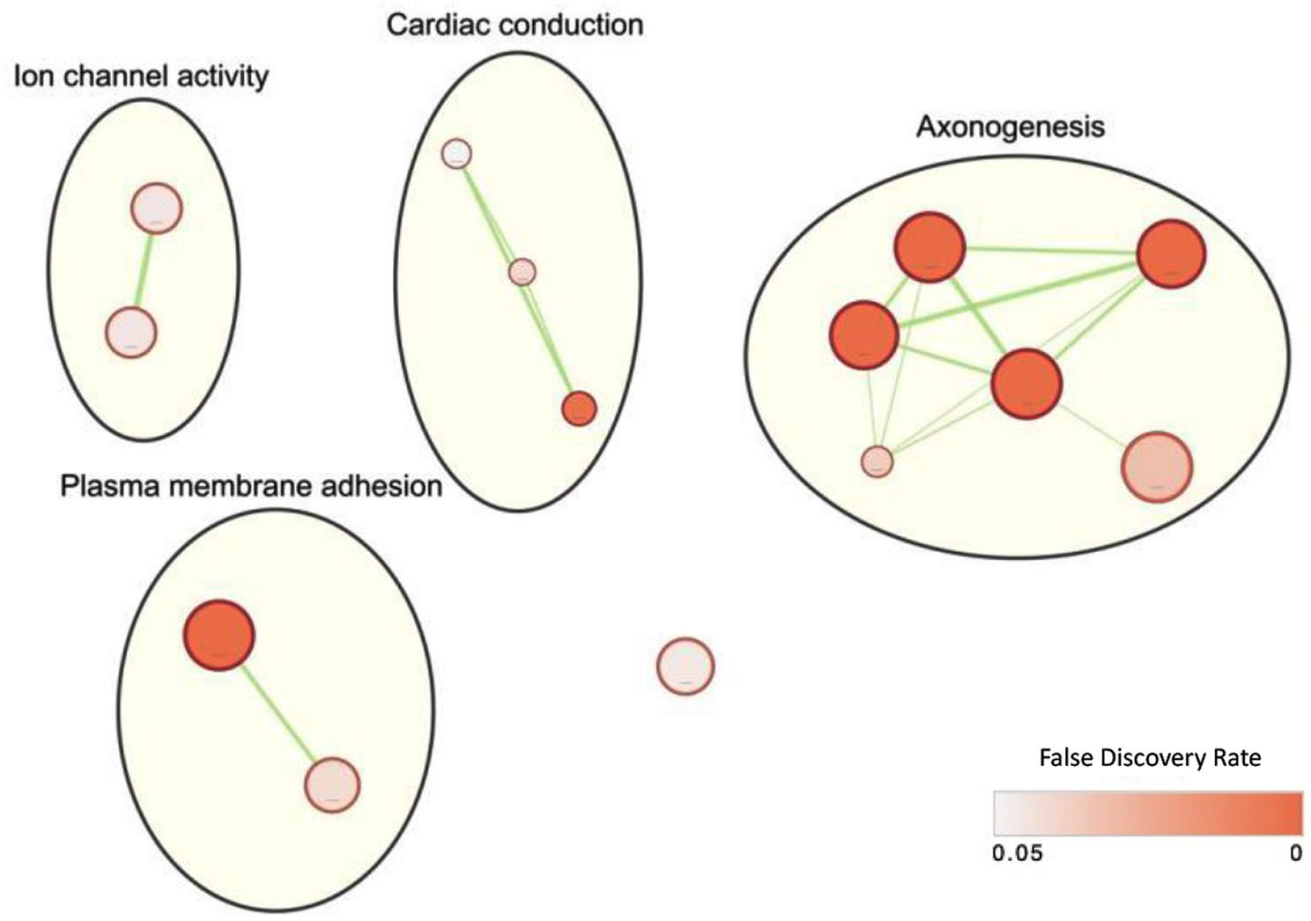
Visualization of gene set enrichment analysis results generated by Cytoscape, using g:Profiler for prioritized genes. Shades of red indicate false discovery rates, calculated based on g:SCS (Set Counts and Sizes, PMID: 17478515). Pathway clustering was carried out with AutoAnnotate, an app in Cytoscape, using the default settings

To functionally characterize the prioritized regions, a comprehensive analysis was conducted on 1,499 coding genes within those regions, including an additional 5 kb upstream and downstream for thorough examination. Gene set enrichment analysis was performed without automated electronic annotations to reveal biological insights. Among the 14 significantly enriched GO annotations with an FDR < 5% (Supplementary Table S2), “Axon Guidance” (GO:0007411) and “Ligand-gated channel activity” (GO:0022834) emerged as the most prominently enriched biological process and molecular function respectively. Clustering of the GO annotations revealed four distinct clusters related to ion channel activity, plasma membrane adhesion, cardiac conduction, and axonogenesis (Fig. 4). Taken together, these findings suggest a potential genetic predisposition in these specific functional areas that may contribute to BNT162b2 vaccine-induced myocarditis.

## Discussion

To the best of our knowledge, this is the first genomic study examining the genetic factors associated with the BNT162b2 vaccinated myocarditis in the largest patient cohort reported. Several epidemiological observations were widely accepted as risk factors associated with the vaccine-related myocarditis. Notable differences were observed in the incidence rate of vaccine-related myocarditis across various age, gender and ethnic group, the underlying reasons were also not fully understood [9]. In addition, long term complications of vaccine-related myocarditis are still not clearly defined. However, impairment of left ventricular and right ventricular myocardial deformation and persistence of late gadolinium enhancement was observed in certain subset of patients up to 1 year after diagnosed with BNT162b2 vaccine-related myocarditis, suggesting the potential long-term effects on the cardiac functions to the affected individuals [33]. By studying the largest representative Chinese vaccine-related myocarditis cohort, further analysis on the genetic background of the susceptible individuals may offer valuable information to identify vulnerable groups within the community and supplementing the explanations for current observed risk factors in the highest risk groups.

The present study offers a preliminary overview of the genetic factors contributing to BNT162b2 vaccine-related myocarditis in susceptible individuals. Specifically, the 2 prioritized clusters overlapped with *BMP3* and *LRRC4C* which was found to be genes involved in inflammation and immunity. *BMP3* codes for an inflammatory protein related to the regulation of the Smad signaling pathway and the release of pro-inflammatory cytokines including IL-6, IL-1β and IL-17A [34]. In the same functional study, *BMP3* inhibition was associated with elevated levels of pro-inflammatory cytokines, suggesting that *BMP3* expression may be involved in inflammation process [34]. Another region (chr11:40,473,809 − 40,585,162) was in the intronic region of Leucine-rich Repeat Containing 4C (*LRRC4C*) gene. *LRRC4C* functions are not directly involved in inflammation, but primarily in immune-related activity such as migration of monocytes, mast cells and M2 macrophages [35]. Moreover, the gene set enrichment analysis in this study provided a broader perspective on the genetic involvement in vaccine-related myocarditis. The identified gene sets in cardiac conduction and ion channel activity suggesting the pre-disposed cardiac functions were also a risk factor in developing the vaccine side effect. A dysfunction of cardiac ion channel and elevated high-sensitivity C-reactive proteins (CRP) was observed in a Brugada syndrome patient that presented with ventricular arrhythmias after receiving BNT162b2 mRNA vaccine, supporting the cardiac ion channel activity may be a potential target in developing the vaccine complications in the susceptible individuals [36]. Apart from that, gene sets related to plasma membrane adhesion revealed an aspect of molecular events that is rarely reported in BNT162b2 vaccine-related myocarditis. The RBD motif in the SARS-CoV-2 spike protein was found eliciting vascular inflammation by binding to the α5β1 integrin in the vascular endothelial cells, in which further demonstrated with systematic production of chemokines and cytokines including TNF-α, IL-1β and IL-6 in the mice model [37]. The molecular mimicry triggered by the spike protein encoded by the vaccine mRNA could elicit similar events but postulated with a possible stronger response to vascular endothelial cells in susceptible individuals. Further investigations are needed in elucidating the possible involvements of the identified genomic clusters and gene sets in BNT162b2 vaccine-related myocarditis. In addition, axonogensis, a biological process extends beyond the current knowledge of vaccine-related myocarditis, showed the most statistical significance in the enriched pathway analysis. Although the crosstalk between axonogensis and BNT162b2 vaccine-related myocarditis has not yet established, this finding may offer a new approach in investigating the vaccine effects and its associated complications.

Findings of this study need to be interpreted with the following caveats. First, due to the rarity of BNT162b2 vaccine-related myocarditis, the results should be interpreted cautiously as the number of patients included in this study was relatively small, although this study is among the largest cohort of BNT162b2 vaccine-related myocarditis reported among adolescents globally. The fast-response and efficient adverse drug reaction reporting system in COVID-19 vaccination and comprehensive clinical management network allowed us to recruit all the confirmed cases within the study period, establishing a BNT162b2 vaccine-related myocarditis patient cohort for further investigation. Second, the choice of controls may not be perfect for our study since the controls were not exposed to mRNA vaccines. We acknowledge this as a limitation of our study and justify that the use of this control cohort is valid as a population control of allele frequencies. Third, the limited analytical power may affect the accuracy of the associations to vaccine-related myocarditis. It is possible that false positives were included during the early analysis stage or due to sequencing errors, which could have influenced the subsequent interpretations. To address these issues, ClusterAnalyzer was utilized to maximize the sensitivity. The focus was primarily on clusters of signals with similar significance, rather than including single discrete signals, in order to minimize the inclusion of false positives. Last, the grouping of SNPs may be inaccurate due to uncertainties in p-value estimates and LD relationships estimates by ClusterAnalyzer. This could have limited the ability of the tool to exclude false positives or to include true positives. The current tool was unable to prioritize genetic variants in the *HLA* and *KIR* loci due to weak LD among variants intrinsic to the hypervariability of these loci. Further modifications should be implemented to enhance the precision and accuracy of the association signals.

## Conclusion

In conclusion, this study has presented preliminary genetic factors associated with BNT162b2 vaccine-related myocarditis. The identified 2,182 genomic clusters potentially associated with the side effect. Further analysis in the genes located in the 2 selected clusters, as well as the enriched pathways analysis, not only align with the current understanding of the BNT162b2 vaccine side effect but also shed light on previously undocumented directions. The group of genes related to inflammation, cardiac conduction, and ion channel activity demonstrated the predisposed immunity and cardiac function differences among the susceptible individuals. Additionally, the highlighted enriched gene sets responsible for plasma membrane adhesion and axonogenesis provided new insights into the genetic factors associated with BNT-162b2 vaccine-related myocarditis. As the vaccine platform is poised to become the foundation for future development of immune protection against pathogens and even disease treatment, it is important to consider identifying individuals who may be more susceptible to major sole-effect of the mRNA platform. This would help mitigate any potential risks associated with mRNA COVID-19 vaccine-related myocarditis.

### Electronic supplementary material

Below is the link to the electronic supplementary material.

Supplementary Material 1

Supplementary Material 2

Supplementary Material 3

Supplementary Material 4

## Data Availability

Source code of ClusterAnalyzer is available on GitHub under BSD-3 license (http://github.com/snakesch/clusterAnalysis). Summary association statistics of raw association signals and prioritized signals were publicly available in the repository.
