# Supplementary figures and images for: Genome-wide association study of BNT162b2 vaccine-related myocarditis identifies potential predisposing functional areas in Hong Kong adolescents
Source: BMC Genom Data. 2024 Jun 6;25:51. doi: 10.1186/s12863-024-01238-6 (PMC11155081; doi:10.1186/s12863-024-01238-6)

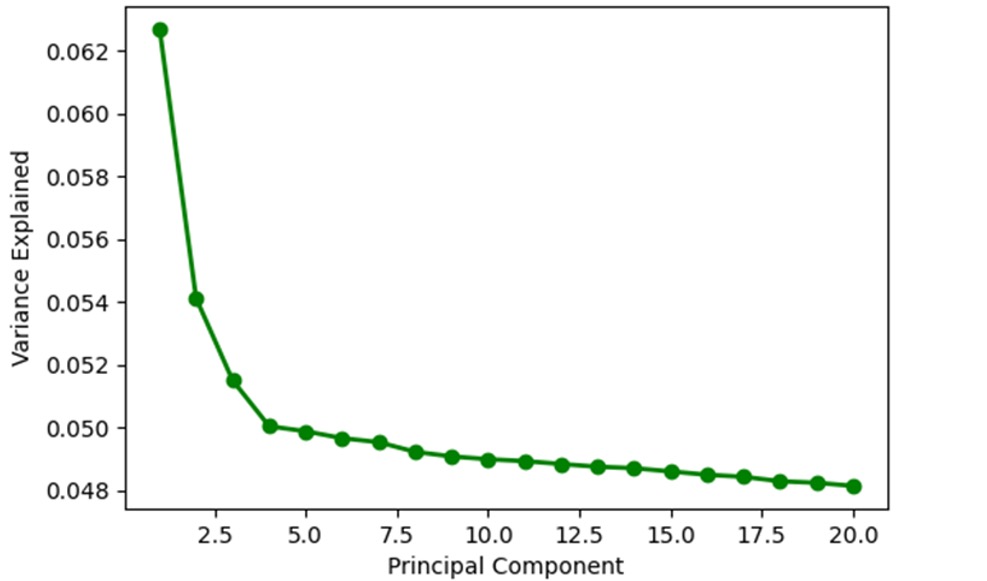

Supplement: Supplementary file 1 — Supplementary Material 1 [file 12863_2024_1238_MOESM1_ESM.jpg]

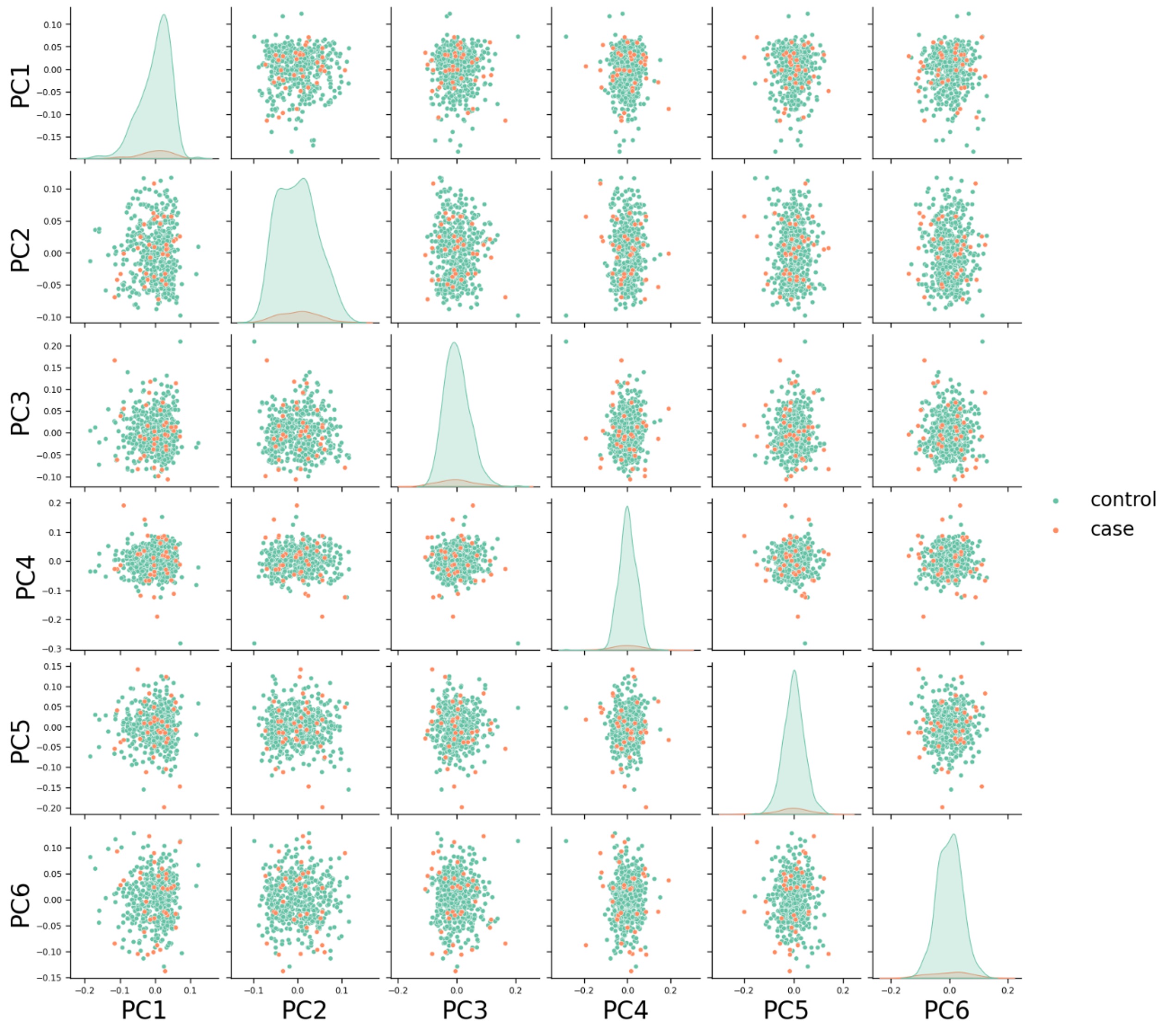

Supplement: Supplementary file 2 — Supplementary Material 2 [file 12863_2024_1238_MOESM2_ESM.jpg]

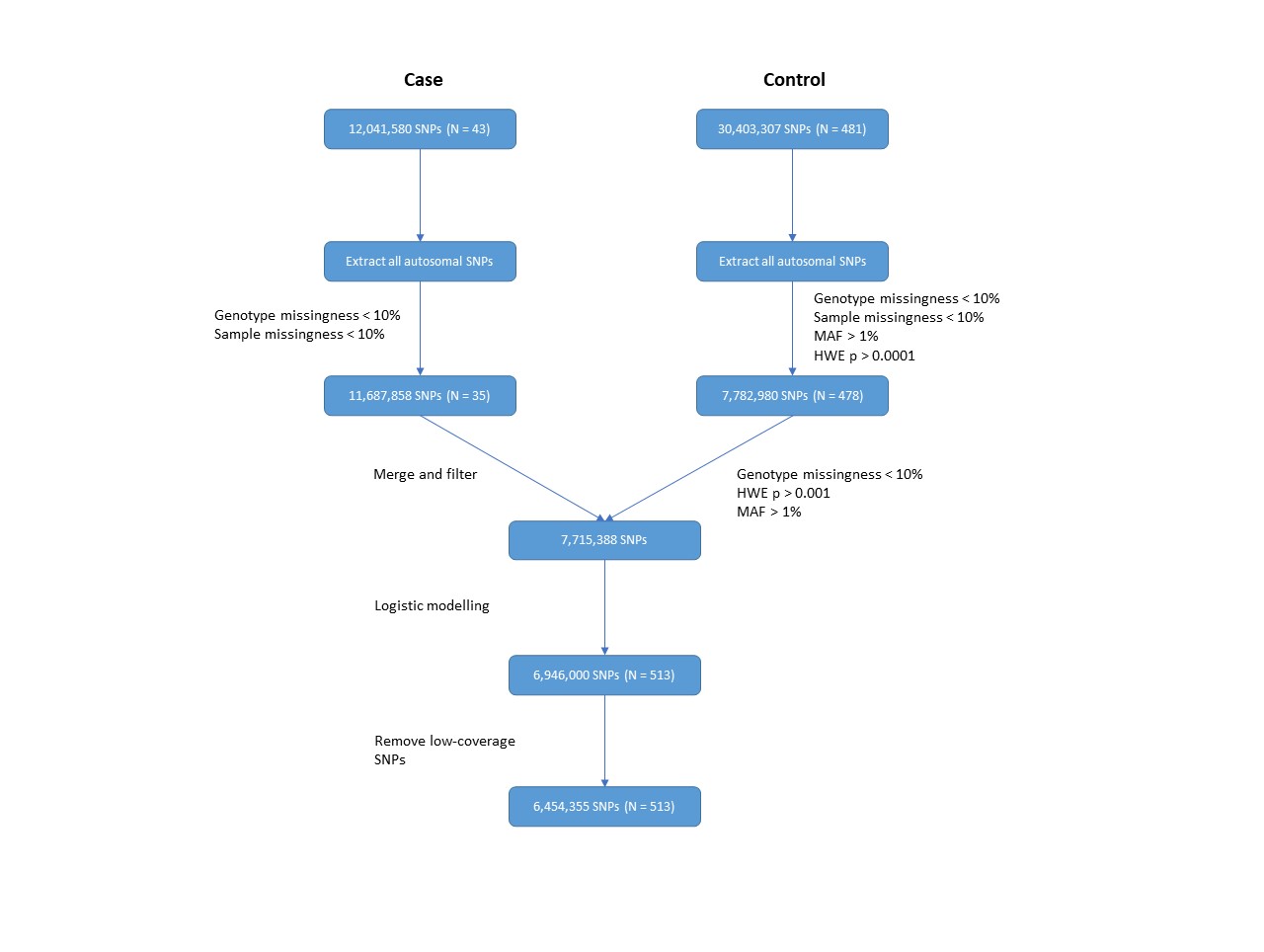

Supplement: Supplementary file 3 — Supplementary Material 3 [file 12863_2024_1238_MOESM3_ESM.jpg]
