# Supplementary material for: Genome-wide association study of BNT162b2 vaccine-related myocarditis identifies potential predisposing functional areas in Hong Kong adolescents
Source: BMC Genom Data. 2024 Jun 6;25:51. doi: 10.1186/s12863-024-01238-6 (PMC11155081; doi:10.1186/s12863-024-01238-6)
Supplement: Supplementary file 4 — Supplementary Material 4 [file 12863_2024_1238_MOESM4_ESM.pdf]

Supplementary Material

Supplementary Data

Figure S1. Overview of quality control steps.

Figure S2. Principal component analysis scree plot.

Figure S3. Principal component analysis biplot.

Table S1. List of prioritized genes (N = 1,499).

Table S2. GO annotations of prioritized genes.

Table S3. Summary statistics table of all SNPs tested for association.

Table S4. Summary of selected clusters (N = 2,182). Two values of SignalCount, Pmin, Pmax are available representing SNPs with OR > 1 and SNPs with OR < 1, respectively.

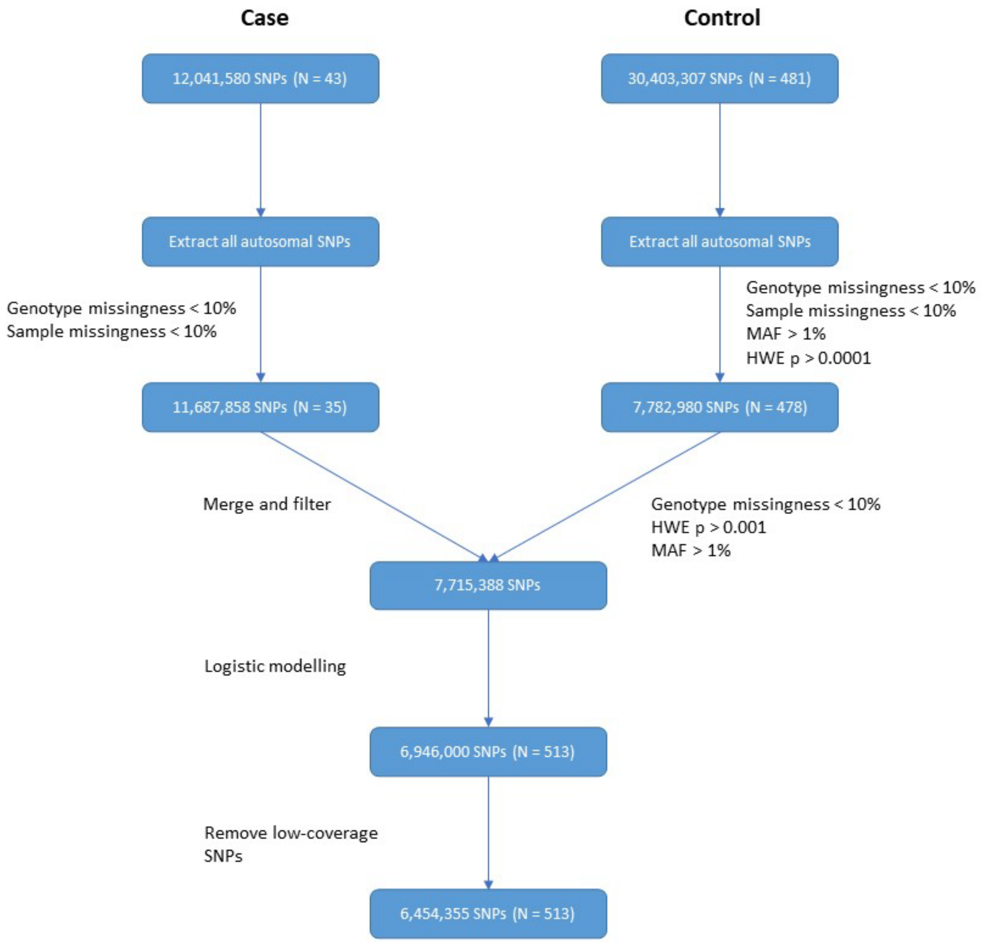

Figure S1. Overview of quality control steps. Number of samples marked in parentheses. HWE: Hardy-Weinberg equilibrium; MAF: minor allele frequency.

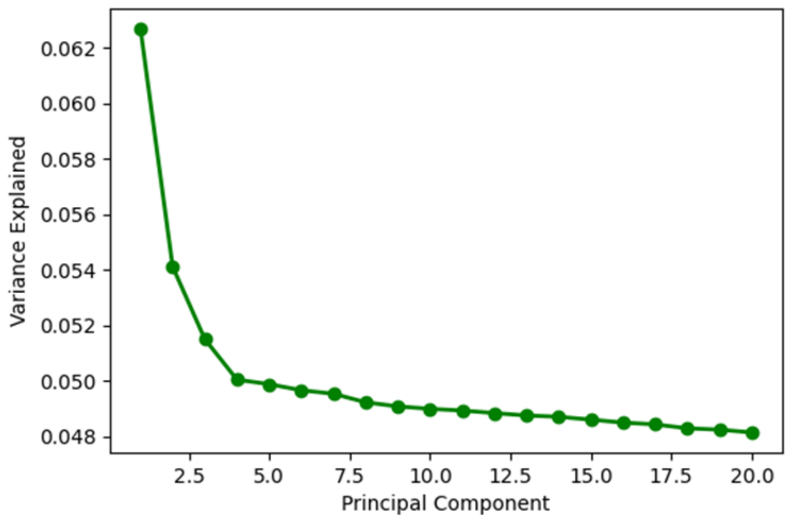

Figure S2. Scree plot showing the percentages of variance explained by each of the 20 principal components. First 6 principal components were included as covariates as the curve plateaued beyond the 6th principal component.

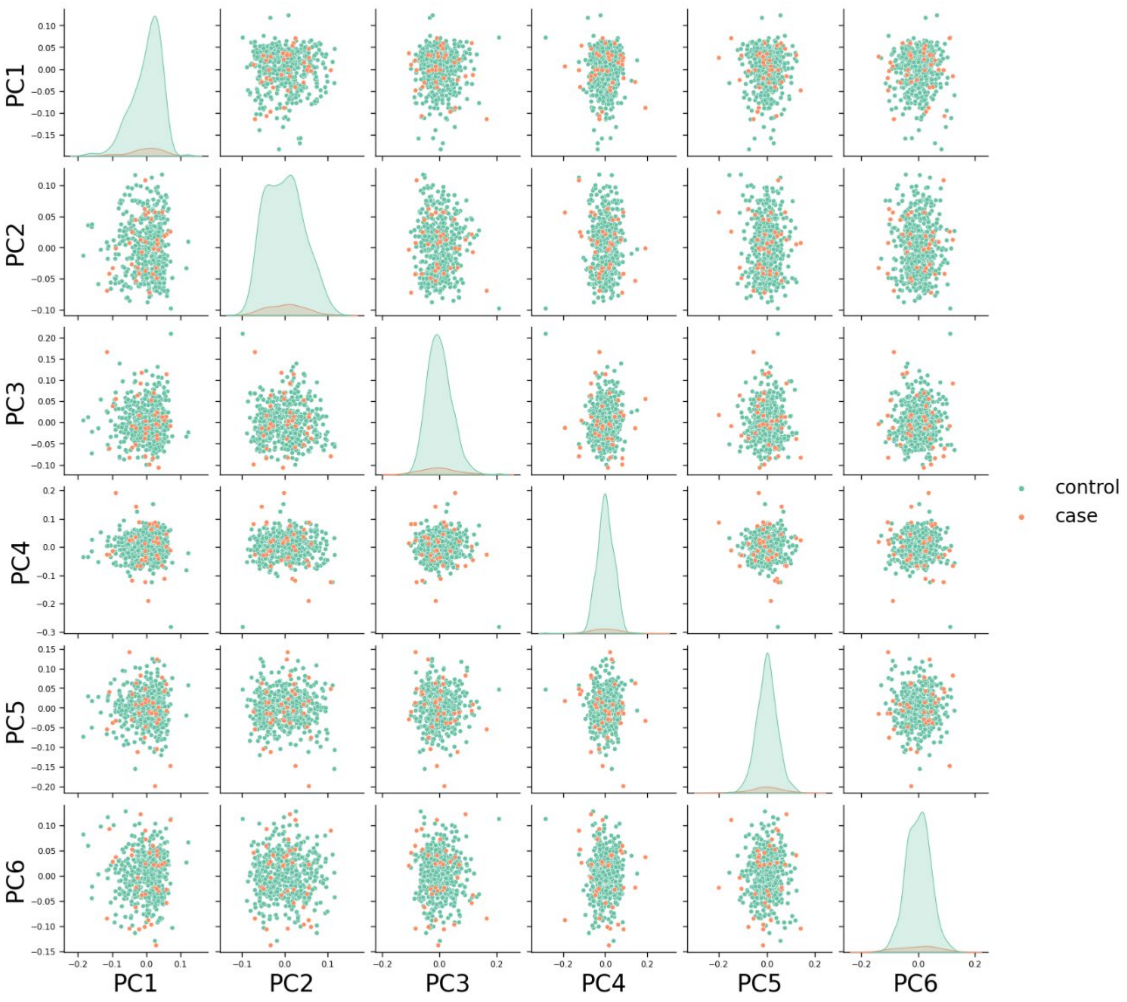

Figure S3. Principal component analysis (PCA) of merged dataset. Only the principal components included as covariates were shown. Green: control; orange: case.
